# Implementations of Evidence-Based eHealth Interventions for Caregivers of People With Dementia in Municipality Contexts (Myinlife and Partner in Balance): Evaluation Study

**DOI:** 10.2196/21629

**Published:** 2021-02-05

**Authors:** Hannah Liane Christie, Lizzy Mitzy Maria Boots, Huibert Johannes Tange, Frans Rochus Josef Verhey, Marjolein Elizabeth de Vugt

**Affiliations:** 1 Department of Psychiatry and Neuropsychology, Alzheimer Centre Limburg School for Mental Health and Neurosciences Maastricht University Maastricht Netherlands; 2 Department of Family Practice Care and Public Health Research Institute Maastricht University Maastricht Netherlands

**Keywords:** eHealth, mHealth, implementation science, dementia, caregiving, municipality

## Abstract

**Background:**

Very few evidence-based eHealth interventions for caregivers of people with dementia are implemented into practice. Municipalities are one promising context in which to implement these interventions due to their available policy and innovation incentives regarding (dementia) caregiving and prevention. In this study, two evidence-based eHealth interventions for caregivers of people with dementia (Partner in Balance and Myinlife) were implemented in 8 municipalities in the Euregion Meuse-Rhine. Partner in Balance is a blended care, 8-week, self-management intervention intervention designed to aid caregivers of people with dementia in adapting to their new roles that is delivered through coaches in participating health care organizations who are trained to use it to offer online support to their clients. Myinlife is an eHealth/mHealth intervention integrated into the Dutch Alzheimer’s Association website and available from the App Store or Google Play, designed to help caregivers of people with dementia use their social network to better organize care and share positive (caregiving) experiences.

**Objective:**

This study’s objectives were to evaluate the success of the implementation of Myinlife and Partner in Balance and investigate determinants of their successful implementation in the municipality context.

**Methods:**

This study collected eHealth use data, Partner in Balance coach evaluation questionnaires, and information on implementation determinants. This was done by conducting interviews with the municipality officials based on the measurement instrument for determinants of implementation (MIDI). These data from multiple sources and perspectives were integrated and analyzed to form a total picture of the determinants (barriers and facilitators to implementation in the municipality context).

**Results:**

The municipality implementation of Partner in Balance and Myinlife showed varying levels of success. In the end, 3 municipalities planned to continue the implementation of Partner in Balance, while none planned to continue the implementation of Myinlife. The 2 Partner in Balance municipalities that did not consider the implementation to be successful viewed the implementation as an external project. For Myinlife, it was clear that more face-to-face contact was needed to engage the implementing municipality and target groups. Successful implementations were linked to implementer self-efficacy and sense of ownership, which seemed to be absent in unsuccessful implementations.

**Conclusions:**

The experiences of implementing these interventions suggested that this implementation context was feasible regarding the required budget and infrastructure. The need to foster sense of ownership and self-efficacy in implementers will be integrated into future implementation protocols as part of standard implementation materials for municipalities and organizations implementing Myinlife and Partner in Balance.

## Introduction

Dementia is a progressive, neurodegenerative disease accompanied by cognitive decline in multiple domains, as well as mood and behavior changes. Informal caregivers play an indispensable role in providing high-quality care for people with dementia [1]. Supporting informal carers of people with dementia is essential, as informal caregiving can potentially allow people with dementia to delay institutionalization and result in positive effects on the person with dementia’s physical and mental health [2]. Given the fact that there are currently 50 million people with dementia worldwide and this number is set to triple by 2050 [3], the rising cost of dementia care and its reliance on informal care is a significant concern for many modern health care systems [4]. Informal caregiving can have both positive [5] and negative [6] effects on the informal caregivers’ physical and mental well-being, and the negative consequences of caregiving can include social isolation, depressive symptoms, stress and anxiety, financial issues, and sleep problems [7,8].

eHealth interventions have been suggested as a means to meet both the demand for more cost-effective dementia health care [9,10] and the need for effective informal caregiver support [11]. Here, eHealth interventions are “treatments, typically behaviorally based, that are operationalized and transformed for delivery via the internet” [12]. Many recent systematic reviews have shown evidence of the effectiveness of eHealth interventions for caregivers of people with dementia, with intervention studies reporting improvements in a variety of caregiver outcomes including increased positive experiences with the caregiving process, self-efficacy, and confidence, in addition to the reduction of stress, experienced burden, and depressive symptoms and anxiety [13-16].

Unfortunately, previous research has shown that very few of these eHealth interventions for caregivers of people with dementia are implemented into practice [17]. Here, implementation refers to “the process of putting to use or integrating evidence-based interventions within a setting” [18]. More generally, only 3% of evidence-based psychosocial interventions for dementia are translated into practice [19]. Lack of proven effects on health care outcomes, doubts from implementing health care staff, meager implementation coordination and management, lack of information on the implementation context, and the fact that users are seldom involved in the eHealth development have been cited as important barriers to the implementation of evidence-based interventions [20-23].

This study was designed to address the lack of information on the implementation context. One potentially important and well-suited implementation context for eHealth interventions for caregivers of people with dementia in Northern Europe is the local municipality. Municipalities are districts or towns with local governments. A municipality’s governing functions can vary from country to country. In general, the municipality is responsible for local services, such as health care, education, recreation, and sport. The municipality context was chosen because municipalities often have policy incentives and funds to address both dementia and caregiving challenges, as well as innovation budgets that are suitable to finance online solutions [24,25]. In this study, two evidence-based eHealth interventions for caregivers of people with dementia (Partner in Balance and Myinlife) were implemented in 8 municipalities in the Euregion Meuse-Rhine (EMR) by municipality officials and by personnel in the local, participating health care organizations. The main research question addressed barriers and facilitators to implementing evidence-based eHealth interventions for caregivers of people with dementia in a municipality context. This study’s specific objectives were to evaluate the success of the implementation of Myinlife and Partner in Balance and investigate determinants of successful implementation of the interventions in the municipality context.

## Methods

### Study Background

**Figure 1:**
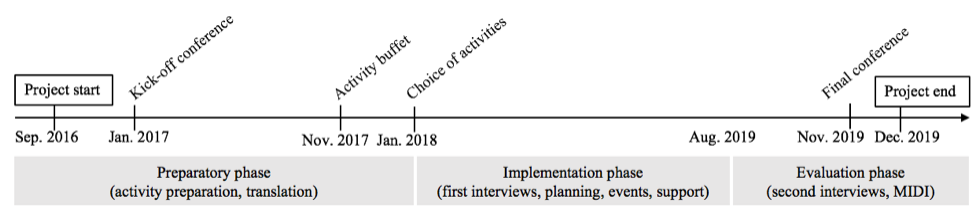
Timeline of eHealth implementation within a Senior Friendly Communities project.

This implementation study took place in the context of the euPrevent Senior Friendly Communities (SFC) project [26], which is based on the World Health Organization’s Active Ageing framework [27]. This project took place between September 2016 and December 2019, and data collection continued until March 2020 (see Figure 1 for a timeline of the project). In this project, 32 municipalities signed up on a first come, first serve basis, with the aim to become more senior-friendly. After a kickoff conference with the participating municipalities and other stakeholders, the project assessed what the municipalities were already doing for their aging population and how they could improve. Informed by this assessment, municipalities selected activities from a so-called activity buffet, which consisted of 15 preexisting activities. These activities were aimed at improving the mental health of the municipality’s aging population by focusing on various aspects of dementia and age-related depression. The activities included a theater production, consultations with experts on various topics, a photo exhibition, courses on relevant topics and psychoeducation, creation and organization of local groups of elderly people, outreach activities, and eHealth interventions to support caregivers of people with dementia. These activities were to be implemented before a final conference with municipalities and stakeholders. Implementation and use of the chosen interventions were included in the participation in the SFC project, meaning that all activities were free of costs for both municipality and users. Data collection took place parallel to the described activities and in 3 phases: preparatory, implementation, and evaluation.

The activity buffet included two eHealth interventions to support caregivers of people with dementia: Partner in Balance and Myinlife. These interventions were included in the activity buffet by the SFC project team due to their promising research results and local origin (they were developed with the EMR). There also was a desire to offer remote support options such as eHealth within the project, and these interventions met this need. Neither had been widely implemented previously, so there were no expectations about which intervention would be easier to implement. Six municipalities opted to implement Partner in Balance (4 in the Netherlands, 1 in Belgium, and 1 in Germany), and 3 opted for Myinlife (2 in Belgium and 1 in Germany). Table 1 depicts relevant characteristics of the SFC municipalities that chose to implement eHealth in their communities. A more detailed description of the municipalities’ eHealth choice process is provided elsewhere [25].

**Table 1:** Characteristics of the participating municipalities^a^.

Characteristics	Value, n
Number of municipalities that chose Partner in Balance	6
Number of municipalities that chose Myinlife	3
Municipality average general population	36,376
Municipality average population age >65 years	7349
Municipality average estimated dementia population	1434

^a^Population statistics sourced from the euPrevent Senior Friendly Communities project [25,26,28].

### eHealth Interventions

#### Partner in Balance

Partner in Balance is an evidence-based eHealth intervention designed to aid caregivers of people with dementia in adapting to their new roles that is delivered through coaches in participating health care organizations who are trained to use it to offer online support to their clients. It is a blended care, 8-week, self-management intervention consisting of (1) an in-person intake session with the coach to acquaint the caregiver with Partner in Balance, select online modules, and set goals; (2) tailored online thematic modules including psychoeducation, behavioral modeling, videos of carers discussing their experiences with the chosen themes, change plans, and email feedback from the coach over 8 weeks; and (3) an in-person evaluation of the program with the coach to assess previously set goals. The in-person meetings between caregiver and coach usually take place at the coach’s place of work (eg, a dementia case management organization) although some coaches choose to visit the caregiver at home. The at-home use of the chosen modules by the caregivers is asynchronous and the responsibility of the caregiver, although the coach provides encouragement and feedback via email. Partner in Balance coaches are required to have experience in health care and dementia care. All coaches take part in a 2-hour Partner in Balance training course with presentation of the intervention and exercises in coaching and self-management techniques. Detailed information about the program components and development is presented elsewhere [29]. Partner in Balance was shown to cause improvements in caregiver outcomes such as mastery, self-efficacy, and quality of life [29,30].

#### Myinlife

Myinlife is an eHealth intervention designed to help caregivers of people with dementia use their social network to better organize care and share positive (caregiving) experiences. Myinlife has been integrated into the Dutch Alzheimer’s Association website [31] and can also be downloaded from the App Store or Google Play free of charge. In previous research, Myinlife has shown potential to make caregiving easier and help caregivers gain more control over their schedules [32,33]. Myinlife has the following functionalities: Profile, Circles, Timeline, Calendar, Helping, Personal Messages, Care Book, and Compass. Caregivers use these functionalities independently (with no help from a coach) to facilitate the organization of care for the person with dementia. Although Myinlife does not make use of a coach, it still requires local health care organizations to facilitate the dissemination and implementation of the intervention by promoting its use among the local population.

### Measures

#### Use Data

Implementation use data was collected for the following measures: number of municipalities choosing one of the interventions, number of research team implementation hours (both face-to-face and remote), number of information technology support hours, and number of accounts (caregivers and coaches). No data were collected on the effect of the intervention or the caregivers’ experiences with the program, as this was assessed in previous research [30].

#### Partner in Balance Coach Evaluation Questionnaire

Because Partner in Balance (but not Myinlife) makes use of a coach as part of its blended approach, evaluation questionnaires were sent to all Partner in Balance coaches who took part in the coach training as part of the SFC project. An English translation of the coach evaluation questionnaire can be found in Multimedia Appendix 1. The questionnaire asked the participants of the training to rate the usability and relevance of Partner in Balance for caregivers and coaches. It consisted of 11 multiple-choice items rated on a 5-point scale (1=completely disagree to 5=completely agree) and 5 open-ended items. A version of this questionnaire had previously been used in the Partner in Balance process evaluation [34].

#### Determinants of Implementation

The measurement instrument for determinants of innovation (MIDI) is designed to assess which determinants may affect implementation, and it can be applied before or after the introduction of an innovation [35]. The MIDI groups determinants into 4 categories: determinants associated with the innovation, adopting person (user), organization, and sociopolitical context. The MIDI was developed to be used in a research context to explore the experiences of intermediary users (“professionals whose actions determine the degree of exposure of end users to the innovation”) of the innovation [36]. To construct the MIDI, determinants were extracted from the results of 8 empirical studies on the implementation of evidence-based innovations and discussed with 22 implementation experts [36]. The instrument consists of 29 questions, each designed to explore a particular determinant. Responses consist of a number on a 1- to 5-point Likert scale and an explanation of the reasoning behind the given score. However, in this study, due to the small sample size, no quantitative MIDI scores were collected, and the MIDI was used instead as a semistructured interview guide to ensure that various domains of implementation were discussed in the evaluation. Multimedia Appendix 2 contains an English version of the MIDI as it was used in these interviews.

### Data Collection

#### Use Data

After each interaction with the municipality, implementation data were anonymously logged in a customized data collection platform with separate entries for each municipality. The interactions included emails, telephone calls, and meetings. The dates and time required for these interactions were logged, including preparations and travel time. Data were logged for all municipalities by author HLC from the start of the implementation in January 2018 until the end of implementation in December 2019.

#### Partner in Balance Coach Evaluation

Coaches were sent the evaluation questionnaire via email at the end of the SFC project in December 2019 and asked to reply via email. Reminders were sent after 6 and 12 weeks. Email responses were stored on the described data collection platform.

#### Determinants of Implementation

Interviews with the municipality representatives responsible for the intervention implementation were conducted to explore determinants of implementation. However, at the end of the project, not all municipalities had achieved the level of implementation necessary to appropriately evaluate implementation determinants using the MIDI questionnaire. The level of eHealth implementation was considered adequate to evaluate determinants if municipalities had completed the implementation activities planned in the initial interviews. These differed per municipality [25] and included a minimum implementation threshold to be considered for determinant assessment. For Myinlife, municipalities must at least have organized caregiver meetings around the intervention. For Partner in Balance, municipalities must have completed a coach training and appointed an organizational Partner in Balance administrator who oversaw the municipalities’ coaches. Implementation levels were assessed prior to the interview by phone by author HLC; 5 municipalities were assessed as having completed the minimum level implementation necessary to conduct an evaluation interview using the MIDI questionnaire as a semistructured interview guide. Interviews were an average of 31 minutes long. For the remaining 3 municipalities, information was collected on the current level of implementation and what steps still needed to be taken via email for one municipality (due to municipality time restraints), via face-to-face meeting for a second, and via telephone meeting for the third.

Interviews occurred between August 2019 and March 2020 and were conducted by author HLC in Dutch, French, or English according to municipality preferences. The MIDI interviews and face-to-face and telephone meetings were recorded and later transcribed verbatim. The written email evaluation was also stored on the data collection platform.

#### Informed Consent and Ethical Approval

All participants (municipality interviewees, Partner in Balance coaches, and experts) had received an information letter explaining the aims of the study, which also guaranteed the anonymous processing of their data and responses, in addition to the option of discontinuing study participation at any point. All participants signed an informed consent form. Ethical approval for the study was granted by Maastricht University’s Medical Ethical Oversight Commission (approval number 2018-0489).

### Data Analysis

#### Use Data

After activities were logged in the online data collection platform by author HLC, total implementation and support hours were automatically calculated across entries and subsequently exported.

#### Partner in Balance Coach Evaluations

Responses were logged in the online data collection platform. Quantitative scores were calculated, and qualitative responses were analyzed inductively by author HLC using analysis software Atlas.ti 8.3 for Macintosh (Atlas.ti Scientific Software Development GmbH). Inductive analysis was used because there were no expectations as to what the open question replies would be. For this analysis, individual codes were independently grouped into themes and categorized by authors HLC and LMMB. Subsequently, HLC and LMMB compared these themes and categories in a consensus meeting with author MEdV to resolve any differences and confirm the final thematic analysis.

#### Determinants of Implementation

Authors HLC and LMMB independently coded the semistructured interviews using deductive thematic analysis [37] in Atlas.ti. In contrast to the open questions in the coach evaluations, it was expected that the interviews would reflect the thematic groups of the consolidated framework for implementation research (CFIR) and not new inductive groups. This is why deductive thematic analysis was used for the interviews. The deductive codes used were CFIR constructs (Table 2). CFIR is an established framework for mapping implementation of evidence-based interventions and can also for used for eHealth interventions [38]. CFIR comprises 5 domains (intervention characteristics, outer setting, inner setting, characteristics of individuals, and process) with 39 implementation constructs. For the analysis, authors LMMB and HLC applied the CFIR codes in Table 2 to interview transcriptions and compared interview segments with the same deductive codes across interviews. Again, HLC and LMMB compared the independently applied codes in a consensus meeting with author MEdV to resolve any differences of opinion. The focus of this analysis was to shed light on the breadth of implementation determinants (barriers and facilitators) in the municipality context.

**Table 2:** Deductive consolidated framework for implementation research codes^a^.

CFIR^b^ domains	Deductive CFIR construct codes
Intervention characteristics	Intervention source
	Evidence strength and quality
	Relative advantage
	Adaptability
	Trialability
	Complexity
	Design quality and packaging
	Cost
Outer setting	Patient needs and resources
	Cosmopolitanism
	Peer pressure
	External policy and incentives
Inner setting	Structural characteristics
	Networks and communications
	Culture
	Implementation climate: Tension for change Compatibility Relative priority Organizational Incentives and rewards Goals and feedback Learning climate
	Readiness for implementation: Leadership engagement Available resources Access to knowledge and information
Characteristics of individuals	Knowledge and beliefs about the intervention
	Self-efficacy
	Individual stage of change
	Individual identification with organization
	Other personal attributes
Process	Planning
	Engaging: Opinion leaders Formally appointed internal implementation leaders Champions External change agents
	Executing
	Reflecting and evaluating

^a^Adapted from Damschoder et al [39].

^b^CFIR: consolidated framework for implementation research.

## Results

### Use Data (Quantitative)

Table 3 shows the use data for Partner in Balance and Myinlife (January 2018 to December 2019). The data show that Myinlife was not chosen a single time in the Netherlands and that Partner in Balance was a more popular choice, especially in the Netherlands. One of the 6 municipalities that initially selected Partner in Balance chose to discontinue the implementation after the first meeting due to a lack of information on future financing and pricing after the project end; this is discussed in depth elsewhere [25]. This municipality is therefore not represented in the table, and averages are calculated over the 5 municipalities that sustained the Partner in Balance implementation. A total of 145 hours were spent on the implementation of Partner in Balance (average 29 hours per municipality), while 54 hours were spent on the implementation of Myinlife (average of 18 hours per municipality).

**Table 3:** Use data by intervention.

Measurement		Partner in Balance	Myinlife
**Number of times implemented by municipalities**			
	Netherlands	3	0
	Belgium	1	2
	Germany	1	1
**Total number of implementation hours (average)**			
	Total remote research team hours	21 (4)	3 (1)
	Total in-person research team hours	124 (25)	51 (17)
Information and communication technology support hours		48	5
**Number of accounts created**			
	Caregivers	22	29
	Coaches	22	—^a^

^a^Not applicable.

### Partner in Balance Coach Evaluations (Quantitative and Qualitative)

Of the 26 coaches who took part in the coach training, only 22 coaches created Partner in Balance coach accounts. An average of 5 coaches were trained per Partner in Balance municipality. Across municipalities, coaches recruited by the municipalities were dementia case managers (7/26), volunteers (3/26), nursing home personnel (6/26), municipality personnel responsible for caregiving (4/26), and dementia outreach nursing staff (6/26). Of the coaches who were sent the coach evaluation questionnaire via email, 64% (14/22) responded, with 57% (8/14) of those (6 Dutch and 2 Belgian coaches) stating they had not been able to use Partner in Balance in their work and thus did not complete the questionnaire. When asked to provide reasons they were not able to begin coaching, 75% (6/8) of those responded: lack of interest from the caregivers in their caseload (n=1), lack of digital skills in caregivers in their caseload (n=1), lack of time to implement the intervention (n=3), and lack of dementia caregivers in their current caseload (n=1), with 2 spontaneously mentioning they found Partner in Balance a very useful and worthwhile tool, despite the barriers. The remaining 43% (6/14) replied with completed questionnaires: 2 from Dutch municipalities, 2 from the German municipality, and 2 from the Belgian municipality.

The results from the completed questionnaires showed that coaches found Partner in Balance to be moderately useful (mean 3.7 [SD 0.8]) and moderately easy to integrate into their jobs (mean 3.3 [SD 0.8]). It was also perceived as a clear added value to the caregiver (mean 4.5 [SD 0.5]) and to the coach, but to a lesser degree (mean 3.5 [SD 0.8]). In general, coaches found it moderately difficult to recruit suitable caregivers (mean 3.5 [SD 1.6)], although this question was not completed by the 2 German coaches. Regarding its advantages for common practice, coaches reported an enriched contact with the caregiver (mean 4.1 [SD 1.0]). They expected the intervention to be time-efficient (mean 4.1 [SD 1.0]) but not cost-efficient (mean 2.8 [SD 1.0]) in the long run. Coaches would recommend Partner in Balance to other care professionals (mean 4.0 [SD 0.9]). Qualitative analysis of the open-ended questions resulted in 2 main findings: lack of digital literacy in the target population and lack of necessary time for the trained coaches to recruit caregivers were perceived as significant barriers.

### Determinants of Implementation (Qualitative)

#### Characteristics of the Intervention

##### Complexity

In general, respondents described Myinife as easy to use. However, one municipality official thought Myinlife was too complicated, as it focused on both online care coordination and positive engagement. This respondent recommended simplifying Myinlife to just the agenda function. Similarly, Partner in Balance was perceived as clear and easy to use. Municipalities found the intervention and coach training easy to understand. However, they would have preferred a more practical, hands-on training in smaller groups, as the training was too theory-focused, and more implementation tips would have been welcome. Also, while Partner in Balance was easy to understand, there were a lot of tasks and organizing involved in making it work (finding coaches, advertising, coordinating, etc), which made it somewhat complex.

##### Design Quality and Packaging

For both interventions, it was reported that more face-to-face meetings and trainings and more advertising and promotional materials were needed. In general, it was suggested that the packaging of the interventions needed to be expanded. For instance, several respondents mentioned that they would like an implementation guidebook. In the current form, coaches receive a guidebook during the training, but the suggested implementation guidebook would help management facilitate the adoption, implementation, and maintenance of the intervention. This would contain a general implementation package, consisting of an implementation protocol and premade templates for social media posts, posters, and flyers.

I think it would really help if you had some kind of general promotion campaign or something, where you have flyers and messages and stuff that you can use. Because now, you really only have the information that is on the flyer on the website, which is actually very similar. And from there you have to figure out everything yourself, and think of messages... While, if you really have posters and flyers and advertising pieces for the local newspapers and such, I think you really already can reach the target group much better.

Municipality R3 (Partner in Balance)

##### Cost

For Partner in Balance, municipalities confirmed that they thought the suggested price model of payment per client was reasonable in theory. The suggested financers were municipalities and advertisers/sponsors. Regarding Myinlife, municipalities liked the idea of clients downloading from the App Store or Google Play, as this seemed to contain less liability for the municipality. In these cases, they suggested price points of €5 ($5.61) and €10 ($11.21). Some respondents also suggested the interventions be free.

##### Relative Advantage

At the end of the implementation, some respondents still preferred face-to-face contact for discussing dementia case management issues. They said that typing sensitive issues on the Partner in Balance platform could be hard for caregivers and coaches, as meanings could be more easily be misconstrued than in face-to-face conversations. Myinlife was considered to be expensive in terms of necessary implementation time compared with having a speaker give a lecture on the topic of dementia caregiving, especially as it is currently impossible for the municipality to see if people are actually using the Myinlife platform. They also wondered if Myinlife really posed an added value compared with other online solutions such as WhatsApp and Facebook. Nevertheless, 28% (9/32) of municipalities in the SFC project chose to implement these eHealth interventions in their communities (although only 8 continued this implementation), indicating that they perceived these interventions as having a relative advantage over the other activities on offer in this project.

#### Characteristics of Individuals

##### Self-Efficacy

Self-efficacy was a recurring topic in the interviews, especially for Partner in Balance, where more guidance of the caregivers and coaches was needed. Both coaches and organization coordinators were uncertain about whether they could fulfill their role and scared to make mistakes. These fears eased once they started the coaching and reported more confidence with increased experience. Municipality officials reported that successful coaches had confidence in the intervention and their own ability to use it to help their clients.

I think that first step was really a big step. But it’s not about saying, “I’m not going to do this.” More, “How do I go about it,” “What is in here?” And from the moment it develops. That’s why I also printed it, had read it, and done all of that while learning, only then did I feel like, okay, now I dare to approach someone with this.

Municipality 4 (Partner in Balance)

##### Knowledge and Beliefs About the Intervention

Municipality officials believed that the interventions would be effective at improving outcomes for caregivers, as this had been proven in previous research which they were familiar with. However, some officials wondered whether these effects would also be obtained outside the research context. For both interventions, there were significant privacy and liability concerns.

I think you should have it in the App Store anyway. And I think that an IT professional from a municipality is really not going to get involved in this, there is also the security issue. If we offer it, and data is lost because you no longer maintain it properly than we are responsible, because we offer it, so I will never get myself into that legal mess.

Municipality 2 (Myinlife)

There were also more general concerns regarding the timeliness and fit of the eHealth interventions in the current dementia health care setting. In particular, they wondered if there was sufficient digital literacy in caregivers, coaches, and in the municipality itself.

#### Inner Setting

##### Structural Characteristics and Networks and Communication

Municipality officials said that much more structural integration was needed. The implementation of the eHealth interventions was usually the sole responsibility of one person within the municipality. Municipality officials stressed that this was not enough, and that there should be a team to tackle the implementation together. As they recommended including this in the product itself, this is discussed in more detail under Characteristics of the Intervention. Municipalities added that it was easy to set up the necessary meetings with the Partner in Balance team.

Look what we can still do is try to launch it in concrete care situations, to see if people use it. But yes, if the guidance is not there, I do not know if they will manage.

Municipality 2 (Myinlife)

##### Implementation Climate

For both interventions, there was not enough goal setting and feedback, interventions had low relative priority, and there were no incentives or rewards to encourage the implementation into clinical practice. As management is primarily interested in concrete output, it is important to keep track of the output and use of the interventions. This is currently possible to track digitally for Partner in Balance but not for Myinlife.

##### Readiness for Implementation

Respondents indicated that there were few resources (especially in terms of available time) to spend on the implementation, as well as a lack of leadership engagement.

#### Outer Setting

##### Cosmopolitanism

Regarding how the implementing organizations are linked to other organizations, respondents stated that the interventions needed to be offered through an external party (not through the municipality) and cooperation with care providers would always be necessary, as they would have to agree to execute the interventions. Some municipalities reported that the SFC project had been a good chance to connect and strengthen their local dementia care networks.

##### Patient Needs and Resources

Myinlife and Partner in Balance were both perceived as fitting caregiver needs. However, for Myinlife, there was little enthusiasm from the local target population, as evidenced by the lack of attendance to the planned Myinlife caregiver meetings.

I think we’ve determined that this should work in principle... But maybe, indeed, it just doesn’t fit what people here want, what they need, what they feel comfortable with. Or maybe we just didn’t reach them despite all the effort... That is also possible.

Municipality 3 (Partner in Balance)

##### External Policy and Incentives

Partner in Balance was described as fitting well into initiatives around generalized services, current internal caregiver and prevention policies, and municipality innovation budgets. These budgets are facilitated by the outer setting, but their use is determined by the inner setting (municipality). The municipalities that had these innovation budgets mentioned that these budgets could potentially be used in the future to purchase licenses for the further implementation of Partner in Balance, if the experiences were positive.

Yes, I think it fits within the policy yes. It fits within the informal care policy, is increasingly in line with the policy of health insurers, who say if we support informal carers then it will yield results. Also for the informal caregiver and the person they care for, so that they stay better in balance, can last longer, so I think it fits within the policy.

Municipality 1 (Partner in Balance)

#### Process

##### Engaging

Municipalities implementing Myinlife indicated that a more hands-on demonstration and sales-pitch–like approach were needed to convince health care partners to cooperate in the dissemination of the intervention and less of an academic presentation. There was not enough engagement of the target populations (both of Partner in Balance coaches and dementia caregivers), although 2 municipalities did involve local dementia groups in their activity choice and subsequent eHealth implementation. More opinion leaders and internal implementation leaders were needed.

I introduced this. My supervisor, yes, but I work in my department alone. .... We have not really discussed it with anyone else. So, my supervisor is not actively pushing this now either.

Municipality 4 (Partner in Balance)

##### Executing

The plans that were made at the beginning of the implementation [25] were followed. Nevertheless, these were in many cases insufficient, and in several municipalities, implementation plans are still being made for the future.

##### Planning

These new plans include involving more local health care groups (for Partner in Balance), more advertising and communications, which are more direct (for both Myinlife and Partner in Balance), and more structural goal setting and feedback (for Partner in Balance, this pertains to coaching; for Myinlife, this is tracking how many people use the intervention). Reflecting and evaluating was not a big part of this implementation but was seen as important for the future implementation of both interventions.

#### Evaluation

Integrating the use data, coach questionnaires, and municipality interviews, it appears that the implementation of Partner in Balance and Myinlife showed varying levels of success in different municipalities. In the end, 3 municipalities planned to continue with their implementation of Partner in Balance beyond the study period, while no municipalities planned to continue with their implementation of Myinlife. What these 3 Partner in Balance municipalities had in common was that they considered the implementation of the intervention to be a success. These municipalities appeared to have a sense of internal responsibility to facilitate the implementation of Partner in Balance and devise creative solutions. The 2 Partner in Balance municipalities that did not consider the implementation to be successful seemed to see the implementation as more of an external project, where the municipality’s role was more to facilitate than execute. For Myinlife, it was clear from the municipality interviews and use data that more time was needed to successfully embed the intervention into the local health care landscape. Despite Myinlife not necessitating the recruitment of coaches, it was clear that more face-to-face contact was needed to engage the implementing municipality and target group.

## Discussion

### Principal Findings

This study integrated use data, coach questionnaires, and interviews to evaluate the implementations of Partner in Balance and Myinlife. These two eHealth interventions for caregivers of people with dementia were implemented in 8 municipalities in the EMR. This study’s objectives were to evaluate the success of the implementation of Myinlife and Partner in Balance and investigate determinants of the successful implementation of Myinlife and Partner in Balance in the municipality context. The analysis of the implementation determinants showed that there were unsuccessful aspects of the implementation, including the lack of goal setting and incentives, low priority, few resources, and lack of leadership. In order to successfully bring evidence-based eHealth interventions for caregivers of people with dementia into practice, a number of important improvements must be made in the implementation of these interventions.

### Improvements for Partner in Balance Coaches

A main finding from the interviews with municipality officials regarding the Partner in Balance implementation was the need to increase the self-efficacy of the Partner in Balance coaches. Coaches reported that uncertainties about whether they were ready to coach and insecurities about whether they could do a good job were significant barriers to starting to coach caregivers. Hence, an important lesson from this study is that Partner in Balance cannot increase caregivers’ self-efficacy without first ensuring that coaches have a minimum level of self-efficacy to start the coaching. This is supported by previous research, which has described care professional self-efficacy as a major facilitator of successful intervention implementation in a variety of contexts [40-42]. Bandura et al [43] described 4 ways to increase self-efficacy: mastery experiences, vicarious experiences, verbal persuasion, and monitoring physiological states. Subsequent research built on this by examining how self-efficacy can be enhanced through training in professional caregivers of people with dementia, which can potentially increase intervention adherence [44]. Discussing common barriers to implementation among the training participants, addressing barriers through role-playing, and providing constructive feedback on the role-play have been shown to increase dementia care professional self-efficacy [45]. In the future, Partner in Balance will incorporate these methods into coach trainings to help coaches develop the self-efficacy necessary to start coaching with Partner in Balance.

In their responses to the request to complete the Partner in Balance coach evaluation questionnaire, several coaches mentioned that they were not able to offer Partner in Balance to any caregivers, as their clients were not familiar with the use of online interventions. These clients were often older, and previous research has indicated that advanced age is a barrier to adopting eHealth due to related declines in motor, cognitive, and perceptive abilities and the difficulties accompanying the rapidly changing technological market [46-48]. In general, studies regarding older adults’ attitudes toward eHealth interventions have produced mixed results [49-51]. It is also important to consider health care professionals’ attitudes toward eHealth for dementia and their role as gatekeepers in deciding whether to offer eHealth interventions such as Partner in Balance to caregivers. In line with this research, a recent systematic literature review on the attitudes of health care professionals toward eHealth described workload concerns, lack of incentives, perceived threats to autonomy, liability concerns, and lack of organizational support and cooperation as important implementation barriers [52]. Here too, a possible remedy for these eHealth challenges experienced by health care professionals is the embedding of improved eHealth education in their standard training [53,54].

### Improvements for Municipalities

For both Myinlife and Partner in Balance, municipality officials reported that their municipality implementation teams were often understaffed. Previous research on municipal eHealth for home care [55] and dementia care [56] has underscored the importance of municipality-specific protocols when implementing eHealth in these contexts. Based on this study, these protocols should specify how to form municipality implementation teams, including suggestions to involve at least 2 people in the team and schedule regular progress meetings within this team. These meetings should discuss new promotion ideas and opportunities using templates for the promotion and advertising of the interventions. Additionally, these meetings should monitor the success of the intervention implementation, as municipality officials reported that their management is most interested in demonstrable output. For Partner in Balance, it is possible for organizations to monitor the number of coaches and participating caregivers. However, there is currently no way to determine whether Myinlife is successfully being used in the community. Previous research on organizational learning as a method for eHealth benefit realization in a municipal health care context emphasized the importance of reviewing and evaluating results and establishing potential for further benefits [57]. This makes it possible for the implementation teams to set and achieve goals around use in the community. In this study, not doing so was counterproductive for both team motivation and acceptability of the time spent on implementation to management. For both Partner in Balance and Myinlife, future implementation packages should include protocols on setting use goals in the regularly scheduled team meetings, and the interventions should include functionalities to easily track these statistics.

### Improvements for Project Management

In order to recruit external health care organizations, the municipality is required to recruit coaches (for Partner in Balance) and integrate interventions into larger health care structures that can offer it as part of their services (for Partner in Balance and Myinlife). This requires regular meetings to follow up on coaches’ experiences, where coaches can learn from each other, share tips and tricks, and discuss their progress. The involvement of the management of these external health care organizations is crucial, as they can offer incentives for successful coaching and adapt structures to facilitate the integration of Partner in Balance into the coaches’ tasks. For example, it is important that management ensures that time spent coaching can be declared to the health insurer as provided care. Previous research has reported this as a significant determinant of successful eHealth implementation for health care professionals [58]. Thus, future implementation packages should include protocols for these organizations on how to organize the suggested meetings, internal monitoring, and incentives, including the declaration of coached hours to health insurers. To facilitate this, future implementation packages should also suggest appointing an eHealth ambassador within the organization whose function is to ensure that these meetings take place, and provide a reliable and continuous level of enthusiasm for the intervention. Previous research has advocated the use of ambassadors in implementing eHealth [32,59-61].

### Sustainability Measures

Despite the relative ease of setting up the infrastructural aspects of this project, implementation was only successful in just over half of the municipalities. It is clear that successful implementation depends on more than merely setting the necessary structures in place. This study’s interview findings indicated that successful implementation was tied to a sense of ownership and responsibility from the municipality officials. This is in line with previous research, which has pointed to a lack of eHealth ownership at both local and national levels as a considerable implementation barrier [62,63]. Therefore, future implementation packages for Myinlife and Partner in Balance will include suggestions on how to achieve sustainability by increasing sense of ownership and end user adherence in general. An important element of this is the reflection and feedback exercises that will also be part of new measures to monitor the interventions (described above), as they have been shown to improve eHealth ownership and adoption [64]. In addition to scheduling the described reflection and role-playing exercises, previous research on increasing the adherence of end users to eHealth interventions recommends persuasive system design, which is used to aid the development of information systems to shape attitudes and behaviors [65]. This approach recommends that interventions incorporate on-the-spot reminders and feedback to increase end user adherence. Hence, future implementations will incorporate more intervention monitoring and reflection moments for implementers and end users. This new approach to training coaches is expected to reduce the uncertainties reported by coaches concerning their abilities to coach.

Finally, it is also important to consider why Partner in Balance was more often successfully implemented in this municipality context than Myinlife. Previous research has indeed shown that blended eHealth interventions for caregivers of people with dementia are more effective at improving outcomes for caregivers of people with dementia than nonblended interventions [13]. One potential explanation for the increased success of Partner in Balance in this particular context is that its blended aspect (the human contact between caregiver and coach) not only increases effectiveness through improved caregiver outcomes but also through a possible effect of increasing engagement among implementers. Here, Partner in Balance required more hours to implement in the municipality context than Myinlife. It is possible that these additional face-to-face hours required to implement Partner in Balance (but not Myinlife) increased implementers’ sense of ownership of the successful implementation of the intervention. Therefore, future implementers of nonblended eHealth interventions in this context could consider incorporating this human interaction by way of face-to-face meetings about the intervention or caregiver support groups discussing the intervention to facilitate implementation by increasing the implementation hours and thus potentially the sense of ownership. Of course, this study also shows that this blended aspect is more resource intensive. Future research could investigate the comparative cost-effectiveness of these interventions in order to weigh costs and benefits.

### Strengths and Limitations

This study had several important strengths. First, this study is one of few to examine the further implementation of eHealth interventions for caregivers of people with dementia after the trial phase. This study uses various measures from multiple perspectives to construct a thorough evaluation of the implementation of these interventions in a municipality context. As a result, this study is able to shed novel light on the currently underexplored organizational and contextual implementation determinants. Second, by focusing on the municipality context specifically and by taking the time to explore this context in depth, this study has successfully identified the municipality as a potential distributor with the financial means to further disseminate evidence-based eHealth interventions for caregivers of people with dementia.

This study also has several limitations. First, this study did not explore the experiences of caregivers using the Partner in Balance and Myinlife interventions. As a result, we have no information on actual eHealth use and do not know how the caregiver target group used and evaluated the interventions in this context. This is because both Partner in Balance and Myinlife were previously assessed for usability and effectiveness by caregivers in a series of trials [29,30,33,66] informed by the Medical Research Council framework [67]. The aim of this study was to gain information on their broader implementation contexts. Second, there was a moderate response rate to the request to complete the Partner in Balance coach evaluation questionnaire (64%), with only 6 coaches submitting completed questionnaires (and 8 providing details on why they had not yet started coaching). As a result, there is no information on how the nonresponders experienced Partner in Balance, causing a potentially biased sample of responses from coaches who might be more positively disposed toward the intervention. Next, this study was unable to take into account the views of the municipalities that chose not to implement Myinlife or Partner in Balance. While it was not this study’s aim to generalize these qualitative findings to all municipalities, it is possible that this study represents a sample of municipalities that have more positive attitudes toward eHealth for dementia and its implementation than other municipalities. Nevertheless, it is still useful to document and learn from these (potentially more engaged) municipalities, as they can provide valuable insight into the feasibility of eHealth for dementia in this context and into municipality needs. Third, the focus of this study was to shed light on the breadth of implementation determinants encountered in bringing evidence-based eHealth interventions for caregivers of people with dementia from research into practice. The aim was to provide a complete overview of the encountered barriers and facilitators using data from a variety of sources. As a result, it must be acknowledged that this study lacks a more elaborate in-depth analysis of the process characteristics of the 8 municipality implementations. Future research will address this topic extensively. Finally, it must be acknowledged that all authors (with the exception of HJT) were involved in the development of Myinlife and Partner in Balance and are therefore potentially not unbiased. However, the authors were also interested in differences between the interventions and were in this sense unbiased. Moreover, it is the authors’ belief that this type of implementation research is essential for evidence-based interventions, and researchers should more often conduct longer term implementation research on their own interventions.

### Conclusions

This study provided a thorough exploration of the feasibility of the implementation of eHealth interventions to support caregivers of people with dementia in a municipality context. Future implementations can make use of protocols that provide municipalities and organizations with suggestions on how to tackle implementation challenges and realize improvements for the (Partner in Balance) coaches, implementation team, and external implementing organizations. In general, it is important to foster a sense of ownership of the success of the eHealth intervention in the municipality and dementia health care context, as this was seen as a main determinant of success in this implementation project. For Partner in Balance, an important finding was that the self-efficacy of coaches must be increased before they can be expected to help caregivers elevate their levels of self-efficacy regarding dementia caregiving. For Myinlife, it was necessary to involve more face-to-face contacts and integrate the intervention more into other local health services, despite it not being designed as a blended intervention. These insights will be integrated into future implementation protocols that will become a standard part of the Myinlife and Partner in Balance implementation packages for municipalities and organizations.

## Appendix

Multimedia Appendix 1 Partner in Balance coach evaluation questionnaire.

Multimedia Appendix 2 Measurement instrument: description and operationalization of determinants.

## Abbreviations

CFIR: consolidated framework for implementation research
EMR: Euregion Meuse-Rhine
MIDI: measurement instrument for determinants of innovation
SFC: Senior Friendly Communities
