# Supplementary material for: Implementations of Evidence-Based eHealth Interventions for Caregivers of People With Dementia in Municipality Contexts (Myinlife and Partner in Balance): Evaluation Study
Source: JMIR Aging. 2021 Feb 5;4(1):e21629. doi: 10.2196/21629 (PMC8081156; doi:10.2196/21629)
Supplement: Multimedia Appendix 1 [file aging_v4i1e21629_app1.docx]

**Appendix 1. Partner in Balance coach evaluation questionnaire**

Some time ago you started as a coach for the online intervention Partner in Balance. We would like to hear from you how you felt about working with Partner in Balance.

This questionnaire consists of a number of closed questions and a number of open questions. You may always give an explanation of your answer, sometimes you will be asked to do so as well.

| Completely disagree | Disagree | Neutral | Agree | Completely agree |
| --- | --- | --- | --- | --- |

1. I think the intervention is useful

for me as a coach. 1 2 3 4 5

2. I can integrate the intervention into

my work. 1 2 3 4 5

3. I think the intervention has an added

value for the caregiver. 1 2 3 4 5

4. I think the intervention has an added

value for the coach. 1 2 3 4 5

5. On average I sept this many hours coaching

for Partner in Balance: ……………….

6. It’s hard to find suitable caregivers to

participate in Partner in Balance. 1 2 3 4 5

7. As a result of using Partner in Balance,

I spend less time on caregiver support. 1 2 3 4 5

Additional question

How many hours more/less?...............

Why?

8. Partner in Balance results in an enriched 1 2 3 4 5

contact with the caregiver

9. Partner in Balance saves time in the long 1 2 3 4 5

Term.

10. Partner in Balance saves costs in the long 1 2 3 4 5

term.

11. I would recommend Partner in balance to 1 2 3 4 5

other care professionals.

12. Did you completed the intervention as planned (1) no + Explanation / (2) yes

(within 9 weeks)?

13. What are the disadvantages to this intervention?

14. What are the advantages to this intervention?

15. What do you think is necessary to implement this intervention within your organisation in the future?

16. I would like to say this in order to improve Partner in Balance in the future:
