# Supplementary material for: Implementations of Evidence-Based eHealth Interventions for Caregivers of People With Dementia in Municipality Contexts (Myinlife and Partner in Balance): Evaluation Study
Source: JMIR Aging. 2021 Feb 5;4(1):e21629. doi: 10.2196/21629 (PMC8081156; doi:10.2196/21629)
Supplement: Multimedia Appendix 2 [file aging_v4i1e21629_app2.docx]

**Appendix 2. Measurement instrument: Description and operationalisation of determinants (adapated from Fleuren et al.[33])**

1 DETERMINANTS ASSOCIATED WITH THE INNOVATION

Determinant 1 Procedural clarity

*Description* Extent to which the innovation is described in clear steps / procedures.

*Operationalisation* The innovation clearly describes the activities I should perform and in which order.

Response scale: (1) totally disagree, (2) disagree, (3) neither agree nor disagree, (4) agree, (5) totally agree

Determinant 2 Correctness

*Description* Degree to which the innovation is based on factually correct knowledge.

*Operationalisation* The innovation is based on factually correct knowledge.

Response scale: (1) totally disagree, (2) disagree, (3) neither agree nor disagree, (4) agree, (5) totally agree

Determinant 3 Completeness

*Description* Degree to which the activities described in the innovation are complete.

*Operationalisation* The innovation provides all the information and materials needed to work with it properly.

Response scale: (1) totally disagree, (2) disagree, (3) neither agree nor disagree, (4) agree, (5) totally agree

Determinant 4 Complexity

*Description* Degree to which implementation of the innovation is complex.

*Operationalisation* The innovation is too complex for me to use.

Response scale: (5) totally disagree, (4) disagree, (3) neither agree nor disagree, (2) agree, (1) totally agree

Determinant 5 Compatibility

*Description* Degree to which the innovation is compatible with the values and working method in place.

*Operationalisation* The innovation is a good match for how I am used to working.

Response scale: (1) totally disagree, (2) disagree, (3) neither agree nor disagree, (4) agree, (5) totally agree

Determinant 6 Observability

*Description* Visibility of the outcomes for the user, for example whether the outcomes of a particular treatment are clear to the user.

*Operationalisation* The outcomes of using the innovation are clearly observable.

Response scale: (1) totally disagree, (2) disagree, (3) neither agree nor disagree, (4) agree, (5) totally agree

Determinant 7 Relevance for client

*Description* Degree to which the user believes the innovation is relevant for his/her client.

*Operationalisation* I think the innovation is relevant for my clients.

Response scale: (1) totally disagree, (2) disagree, (3) neither agree nor disagree, (4) agree, (5) totally agree

**2. DETERMINANTS ASSOCIATED WITH THE USER**

Determinant 8 Personal benefits/drawbacks

*Description* Degree to which using the innovation has advantages or disadvantages for the users themselves.

*Operationalisation* To what extent does using the innovation have personal benefits/drawbacks for you?

This question is asked for each concrete benefit or drawback that is expected to be salient for the particular user population.

Response scale advantages: (1) totally disagree, (2) disagree, (3) neither agree nor disagree, (4) agree, (5) totally agree

Response scale disadvantages: (5) totally disagree, (4) disagree, (3) neither agree nor disagree, (2) agree, (1) totally agree

Determinant 9 Outcome expectations

*Description* Perceived probability and importance of achieving the client objectives as intended by the innovation

*Operationalisation* Composite measure: the product of *importance* and *probability*

These questions about the importance and probability are asked for each objective separately.

*Importance*

I think it is important to achieve the following objectives for my client …[state objectives].

Response scale: (1) totally disagree, (2) disagree, (3) neither agree nor disagree, (4) agree, (5) totally agree

*Probability*

I expect that using the innovation will actually achieve the following objectives for my client …[state objectives].

Response scale: (1) most definitely not, (2) definitely not, (3) perhaps not, perhaps, (4) definitely, (5) most definitely

Determinant 10 Professional obligation

*Description* Degree to which the innovation fits in with the tasks for which the user feels responsible when doing his/her work.

*Operationalisation* I feel it is my responsibility as a professional to use this innovation.

This question is asked for each activity in the innovation.

Response scale: (1) totally disagree, (2) disagree, (3) neither agree nor disagree, (4) agree, (5) totally agree

Determinant 11 Client satisfaction

*Description* Degree to which the user expects clients to be satisfied with the innovation.

*Operationalisation* Clients will generally be satisfied if I use this innovation.

Response scale: (1) totally disagree, (2) disagree, (3) neither agree nor disagree, (4) agree, (5) totally agree

Determinant 12 Client cooperation

*Description* Degree to which the user expects clients to cooperate with the innovation.

*Operationalisation* Clients will generally cooperate if I use this innovation.

Response scale: (1) totally disagree, (2) disagree, (3) neither agree nor disagree, (4) agree, (5) totally agree

Determinant 13 Social support

*Description* Support experienced or expected by the user from important social referents relating to the use of the innovation (for example from colleagues, other professionals they work with, heads of department or management).

*Operationalisation* I can count on adequate assistance from my colleagues if I need it to use the innovation.

This question is asked for important social referent group or person inside or outside the organisation (colleagues, immediate hierarchical superior, management, professionals involved in the delivery of care etc.).

Response scale: (1) totally disagree, (2) disagree, (3) neither agree nor disagree, (4) agree, (5) totally agree

Determinant 14 Descriptive norm

*Description* Colleagues' observed behaviour; degree to which colleagues use the innovation.

*Operationalisation* In your opinion, what proportion of the colleagues in your organisation for whom the innovation is intended actually use the innovation?

Response scale: (1) not a single colleague (2) almost no colleagues (3) a minority (4) half (5) a majority (6) almost all colleagues (7) all colleagues.

Determinant 15 Subjective norm

*Description* The influence of important others on the use of the innovation.

*Operationalisation* Composite measure: the product of *normative beliefs* and *motivation to comply.* These questions about normative beliefs and motivation to comply are asked for each referent person/group inside or outside the organisation (colleagues, heads of department, management, clients etc.).

*Normative beliefs*

To what extent do the following people [list people] expect you to use the innovation?

Response scale: (1) most definitely not (2) definitely not (3) perhaps not, perhaps (4) definitely (5) most definitely

*Motivation to comply*

When it comes to working in accordance with the innovation, to what extent do you comply with the opinions of the following people [list people]?

Response scale: (1) very little (2) little (3) not a little, not a lot (4) a lot (5) a great deal

Determinant 16 Self-efficacy

*Description* Degree to which the user believes he or she is able to implement the activities involved in the innovation.

*Operationalisation* Should you wish to do so, do you think you can put [state activity from the innovation] into practice?

This question is asked for each activity in the innovation.

Response scale: (1) most definitely not (2) definitely not (3) perhaps not, perhaps (4) definitely (5) most definitely

Determinant 17 Knowledge

*Description* Degree to which the user has the knowledge needed to use the innovation.

*Operationalisation Objective measurement* with a knowledge test including a range of questions.

*Subjective measurement* with one question:

I know enough to use the innovation.

Response scale: (1) totally disagree, (2) disagree, (3) neither agree nor disagree, (4) agree, (5) totally agree

Determinant 18 Awareness of content of innovation

*Description* Degree to which the user has learnt about the content of the innovation.

*Operationalisation* To what extent are you informed about the content of the innovation?

Response scale: (1) I'm not familiar with the innovation (2) I'm familiar with the innovation, but I haven't read it through (yet) (3) I'm familiar with the innovation and I've glanced through it (4) I'm familiar with the innovation and I have read through it thoroughly

**3. DETERMINANTS ASSOCIATED WITH THE ORGANISATION**

**Determinant 19 Formal ratification by management**

*Description* Formal ratification of the innovation by management, for example by including the use of the innovation in policy documents.

*Operationalisation* Has the management set up formal arrangements in your organisation relating to the use of this innovation (in policy plans, work plans and so on)?

Response scale: (1) no (2) yes

Determinant 20 Replacement when staff leave

*Description* Replacement of staff leaving the organisation

*Operationalisation* In my organisation, there are arrangements in place so that staff who use the innovation and leave the organisation are replaced in good time by employees who are/will be adequately prepared to take over.

Response scale: (1) totally disagree, (2) disagree, (3) neither agree nor disagree, (4) agree, (5) totally agree

Determinant 21 Staff capacity

*Description* Adequate staffing in the department or in the organisation where the innovation is being used.

*Operationalisation* There are enough people in our organisation to use the innovation as intended. Response scale: (1) totally disagree, (2) disagree, (3) neither agree nor disagree, (4) agree, (5) totally agree

Determinant 22 Financial resources

*Description* Availability of financial resources needed to use the innovation.

*Operationalisation* There are enough financial resources available to use the innovation as intended. Response scale: (1) totally disagree, (2) disagree, (3) neither agree nor disagree, (4) agree, (5) totally agree

Determinant 23 Time available

*Description* Amount of time available to use the innovation.

*Operationalisation* Our organisation provides me with enough time to include the innovation as intended in my day-to-day work.

Response scale: (1) totally disagree, (2) disagree, (3) neither agree nor disagree, (4) agree, (5) totally agree

Determinant 24 Material resources and facilities

*Description* Presence of materials and other resources or facilities necessary for the use of the innovation as intended (such as equipment, materials or space).

*Operationalisation* Our organisation provides me with enough materials and other resources or facilities necessary for the use of the innovation as intended.

Response scale: (1) totally disagree, (2) disagree, (3) neither agree nor disagree, (4) agree, (5) totally agree

Determinant 25 Coordinator

*Description* The presence of one or more persons responsible for coordinating the implementation of the innovation in the organisation.

*Operationalisation* In my organisation, one or more people have been designated to coordinate the process of implementing the innovation.

Response scale: (1) no (2) yes

Determinant 26 Unsettled organisation

*Description* Degree to which there are other changes in progress (organisational or otherwise) that represent obstacles to the process of implementing the innovation, such as re- organisations, mergers, cuts, staffing changes or the simultaneous implementation of different innovations.

*Operationalisation* Are there, in addition to the implementation of [describe innovation], any other changes in the organisation affecting the implementation of the innovation now or in the foreseeable future (reorganisation, merger, cuts, staffing changes, other innovations)?

Response scale: (2) no (1) yes

Determinant 27 Information accessible about use of innovation

*Description* Accessibility of information about the use of the innovation.

*Operationalisation* It is easy for me to find information in my organisation about using the innovation as intended.

Response scale: (1) totally disagree, (2) disagree, (3) neither agree nor disagree, (4) agree, (5) totally agree

Determinant 28 Performance feedback

*Description* Feedback to the user about progress with the innovation process.

*Operationalisation* In my organisation, feedback is regularly provided about progress with the implementation of the innovation.

Response scale: (1) totally disagree, (2) disagree, (3) neither agree nor disagree, (4) agree, (5) totally agree

**4. DETERMINANTS ASSOCIATED WITH THE SOCIO-POLITCAL CONTEXT**

**Determinant 29 Legislation and regulations**

*Description* Degree to which the innovation fits in with existing legislation and regulations established by the competent authorities (examples being financial structures, or substantive legislation and supervision from the Dutch Health Care Inspectorate or the Dutch Care Authority).

*Operationalisation* The activities listed in the innovation fit in well with existing legislation and regulations.

Response scale: (1) totally disagree, (2) disagree, (3) neither agree nor disagree, (4) agree, (5) totally agree.

**5. OTHER QUESTIONS**

Have we followed the implementation plan? Why (not)?

Which parts of this plan worked, which parts did not work?

Have we forgotten anything in this plan / what could be improve?
